# The Psychological Impact of Dealing with Death and the Risk of Dying Among Nurses Working in ICU and NICU: Specificities in Mediating and Moderating Variables

**DOI:** 10.3390/healthcare13182265

**Published:** 2025-09-10

**Authors:** Federica Vallone, Carmine Vincenzo Lambiase, Maria Clelia Zurlo

**Affiliations:** 1 Department of Humanities, University of Naples Federico II, 80133 Naples, Italy; federica.vallone@unina.it (F.V.); carminevincenzo.lambiase@unina.it (C.V.L.); 2 Dynamic Psychology Laboratory, University of Naples Federico II, 80133 Naples, Italy

**Keywords:** comparative study, coping strategies, death and dying, intensive care unit, mediating and moderating variables, neonatal intensive care unit, psychological health, risk and protective factors, stress, work-resources

## Abstract

**Background/Objectives**. This study applied the *Demands-Resources-and-Individual-Effects(DRIVE)-Nurses-Model* to explore and compare the experiences of nurses working in Intensive Care Units (ICUs) and in Neonatal Intensive Care Units (NICUs), by investigating the effects of the interplay (main/mediating/moderating effects) of perceived stress related to dealing with death/critically ill patients (*Death-and-Dying-Stressor*)—which unavoidably features in the daily life of nurses working in ICU/NICU—with further potential *Stressors in Nursing* (Conflicts-with-Physicians, Peers, Supervisors, Patients/their families, Uncertainty-Concerning-Treatment, Inadequate-Emotional-Preparation, Discrimination, Workload), *Work-Resources* (Job-Control, Social-Support, Rewards), and *Coping-Strategies* (Problem-focused, Seek-Advice, Self-Blame, Wishful Thinking, Escape/Avoidance) on nurses’ psychological health conditions according to the working unit (ICU/NICU). **Methods**. Overall, 62 critical care nurses (ICU = 35; NICU = 27) completed self-report questionnaires. Main/mediating/moderating effects were tested by using Correlational-Analyses and Hayes-PROCESS-tool by working unit. **Results**. Nurses working in NICU reported higher *Psychological Disease* than nurses working in ICU. The detrimental psychological impact of *Death-and-Dying-Stressor* was mediated by *Conflicts-with-Supervisors*-*Stressor* among ICU nurses and by *Uncertainty-Concerning-Treatment* and *Conflicts-with-Physicians* stressors among NICU nurses. The recourse to *Self-Blame* and *Escape/Avoidance* coping strategies exacerbated the psychological risk among ICU nurses, while perceived *Work-Resources* (Job-Control/Social-Support) played a protective moderating role among NICU nurses. **Conclusions**. The application of the *DRIVE-Nurses-Model* to deepen the experience of nurses working in ICU/NICU could advance the understanding of the mechanisms underlying the relationship between *Death-and-Dying-Stressor* and nurses’ psychological health, suggesting tailored risk profiles and accounting for key protective factors, to provide nurses with the necessary resources for adjusting to their challenging and emotionally demanding work-related duties and experiences.

## 1. Introduction

Nursing is increasingly regarded as one of the most challenging and emotionally demanding occupations [1,2,3], and this is particularly true for nurses working in Intensive Care Units (ICUs) [4,5,6,7,8], who are indeed required not only to provide highly qualified medical assistance, but also to manage—often without any training or support—the emotional burden of dealing with patients under life-threatening conditions along with the emotional load of patients’ relatives [9,10,11].

From this perspective, research has clearly demonstrated that nurses’ duties and tasks—both the formal and the informal (relational) ones—continuously challenge them with a wide set of specific stressors [12,13], such as high workload [14], perceived inadequate emotional and technical preparation [15,16,17], along with the management of relationships/potential conflicts within the wards, i.e., with supervisors, with/between nurses, physicians, and patients and their relatives [18].

However, above all, research highlighted the emotional demands of dealing with death and dying/suffering patients as being one of the main stressors threatening nurses’ wellbeing [5,15,16]. Dealing with death and dying/suffering patients is—indeed—the sole unavoidable source of stress repeatedly and inherently featuring the daily life of nurses [13,19], “silently” threatening their psychological health conditions repeatedly over each work-shift, beyond the working unit. This, however, can be particularly/differently challenging in ICU, in which nurses deal with critically ill, unstable, and/or dying adult patients [10,20,21,22,23,24] and, even more, in Neonatal Intensive Care Units (NICUs) [25,26,27], where it is a potential and very fragile new life that is ‘suspended’, and nurses are also required—to a greater extent—to take charge of the emotional containment (fears/hopes) of the patient’s relatives. From this perspective, the requests of dealing with the fragility of newborn babies in life-threatening/unstable conditions (often for a prolonged period)—along with the requests for information and reassurances from the family members—could potentially exacerbate the burden of NICU nursing staff, who need to reckon with the reality that—despite all the efforts and the rigorous following of the “gold standard” of care—the newborn could not survive.

Despite the clear psychological costs of dealing with death and dying patients in ICU [10,20,21,22,23,24] and NICU [25,26,27], there is still a lack of studies that explore—from a comprehensive and more complex perspective—the psychological impact of this stressor, namely by also considering the role of further factors potentially intervening in this well-demonstrated relationship. In other words, there is a need to develop research aimed at understanding the mechanisms underlying the relationship between *Death-and-Dying-Stressor* and nurses’ psychological health conditions by identifying further and—most importantly—modifiable factors that can exacerbate or mitigate the detrimental effects of this unmodifiable stressor. This could indeed support the development of targeted interventions fostering the psychological health of nurses working in ICU and NICU [9], who need to be provided with adequate resources for adjusting to highly demanding work duties and the routine experiences with death and the dying.

In this direction, recently, research has provided a statistically valid multidimensional transactional model, namely the *Demands Resources and Individual Effects* (*DRIVE*)-*Nurses-Model* [28,29,30]. This model allows researchers and practitioners to assess and monitor the impact of a wide range of factors influencing nurses’ psychophysical health conditions. This is by accounting for the effects of the complex interplay among risks and resources (i.e., exploring and identifying main/mediating/moderating effects), reflecting real-life circumstances in which nurses are simultaneously exposed to multiple hazards yet they could also possess different resources to deal with them [28,29,30]. In this perspective, previous research applications of the *DRIVE-Nurses-Model* underlined risk profiles and vicious circles undermining nurses’ psychological health, yet also identifying specific coping strategies [30,31], along with work-resources (i.e., job control, social support, rewards), that are able to significantly counteract the detrimental effects of risk factors on nurses’ wellbeing [29,30,31,32,33].

Therefore, it can be hypothesized that the application of the *DRIVE-Nurses-Model* to improve the experience of nurses working in ICU and NICU would also advance the understanding of the mechanisms underlying the relationship between the main and unavoidable stressor of *Death-and-Dying* and nurses’ psychological health conditions by identifying further risk and protective factors (i.e., *Stressors in Nursing*, *Work-Resources* and *Coping-Strategies*) intervening in this association and potentially targeting them through tailored interventions.

The present study proposed a research application of the *DRIVE-Nurses-Model* [28,29,30,31,32,33] to achieve a more comprehensive understanding of the experience of nurses working in ICU and NICU, with a particular focus on the psychological impact of dealing with death/critically ill/unstable patients. Specifically, this study aims to firstly explore and compare the experiences of nurses working in ICU and NICU by investigating the effects of the interplay (main/mediating effects) between the specific and unavoidable *Stressor in Nursing* of *Death-and-Dying* with further potential *Stressors in Nursing* (Conflicts-with-Physicians, Peers, Supervisors, Patients/their families, Uncertainty-Concerning-Treatment, Inadequate-Emotional-Preparation, Discrimination, Workload) on nurses’ psychological health conditions, according to the working unit (ICU and NICU). Also, given the interest in identifying potential individual and work-related resources able to further intervene in this association, the potential moderating role of *Coping-Strategies* (Problem-focused, Seek-Advice, Self-Blame, Wishful Thinking, Escape/Avoidance) and *Work-Resources* (Job-Control, Social-Support, Rewards) was explored and compared according to the working unit.

**Figure 1:**
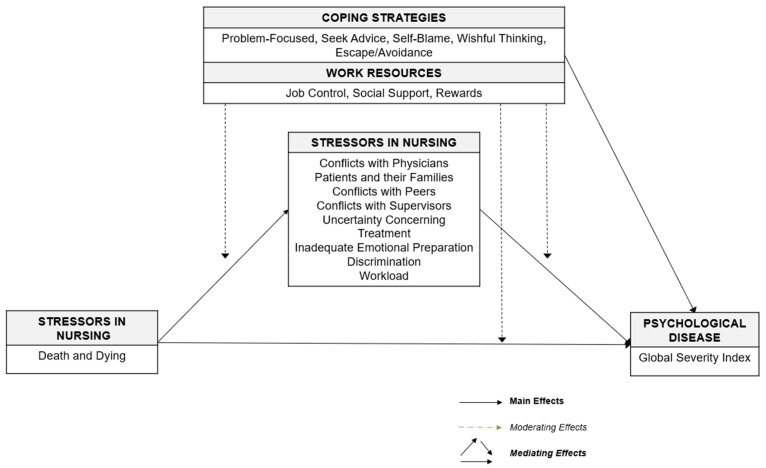
Conceptual framework.

In line with the study aims, the following research questions have been developed, graphically illustrated (Figure 1), and originally tested:

*Research Question One (RQ1)—Preliminary Differences in Study Variables*: Are there differences in Background Information, in perceived levels of Stressors in Nursing, in the recourse to Coping Strategies, and in perceived levels of Work-Resources and Psychological Disease reported by nurses according to the working unit (ICU and NICU)?

*Research Question Two (RQ2)—Main Effects:* Are there differences in the associations between perceived *Stressors in Nursing*, *Coping-Strategies*, and *Work-Resources* respectively, with *Psychological Disease* according to the working unit (ICU and NICU)?

*Research Question Three (RQ3)—Mediating Effects*: Is the relationship between Death-and-Dying-Stressor and Psychological Disease mediated by further Stressors in Nursing across the two working units (ICU and NICU)?

*Research Question Four (RQ4)—Moderating Effects*: Do Coping-Strategies and Work-Resources serve as significant moderators of the relationships between Death-and-Dying-Stressor, further Stressors in Nursing, and Psychological Disease, respectively, across the two working units (ICU/NICU)?

## 2. Materials and Methods

### 2.1. Design and Participants

This cross-sectional study is reported in accordance with the EQUATOR Network-guidelines (STROBE-checklist). The study was conducted in a sample of sixty-two nurses working in Intensive Care Units (ICU and NICU), recruited from five Hospitals of the Public Health Service in Southern Italy between January 2024 and October 2024, as part of a larger project targeting *Stress-in-Nursing* [29,30,31,32,33]. Preliminarily, chairpersons/managers were asked to provide permission for proposing the survey to the staff. Therefore, nurses were approached by a trained psychologist—always available during the paper-and-pencil in-person administration to answer any doubts/questions and to support the completion of the questionnaire—and nurses were provided with a standardized introduction of the project and with informed consent. To be included in the study, nurses needed to work in ICUs of the Italian Public Health Service. Those working in different wards were not included in this study. Overall, sixty-two nurses working in ICUs provided informed consent and completed the survey. The total sample comprised both men (40.3%) and women (59.7%). Also, the sample covered staff members of the nursing workforce representative of different ages/stage careers, from the younger and newly enrolled nurses to the elderly/more experienced nurses (Age *Mean* = 42.9, *SD* = 9.9; Range = 23–61 years). There was no missing data. The study was approved by the Ethics Committee of Psychological Research of University of Naples Federico II (Protocol Number: 33/2019).

### 2.2. Measures

The survey comprised a section for assessing nurses’ background information, along with validated measures for the assessment of perceived *Stressors in Nursing* [34], *Work-Resources* [35,36,37], *Coping-Strategies* [38], and *Psychological Disease* [39,40]. Table 1 illustrates the description of measures.

### 2.3. Analytical Plan

Firstly, to address *RQ1 (Differences in Study Variables*), descriptive statistics were computed, and cross-tabulations (χ^2^) and Student’s *t*-test analyses were used to compare study variables according to the working unit (ICU and NICU). To judge the normality of data, the distribution of variables was explored by calculating Skewness and Kurtosis values; i.e., Skewness ±2 and Kurtosis ±7 were considered to be a violation of normality [41,42,43,44]. Also, for study variables, Cohen’s *d* (along with 95% Confidence Interval) was used to inspect the effect sizes, with *d* values considered as small (*d* = 0.2), medium (*d* = 0.5), and large (*d* = 0.8) in line with Cohen’s rule of thumb.

Furthermore, for *Psychological Disease,* in order to clinically interpret the levels of psychological burden, frequencies and percentages of nurses reporting low (below the cut-off point scores) and high (clinically relevant) levels of psychopathological symptoms were also calculated (and compared according to the working unit) by using the cut-off scores for the Global Severity Index (GSI) provided by the Italian validation study of the SCL-90-R [40], namely 0.97 for men and 1.24 for women, respectively. Since *RQ1* involves multiple comparisons, the Bonferroni–Holm correction method was used to adjust the *p*-values (α = 0.05), thus reducing the risk of Type I error.

Secondly, to address *RQ2 (Main Effects*), as well as to evaluate the feasibility of the testing of mediating and moderating analyses, Pearson’s correlations among study variables were conducted. Afterwards, correlations (Spearman’s rank correlation coefficient) between *Background Information* (Sex; Age; Working Seniority; Type of Contract; Night Shifts) and study variables were also conducted to verify whether background factors should be included as control variables in mediating and moderating analyses.

Therefore, to address *RQ3 (Mediating Effects*), Model 4 from Hayes-PROCESS-tool was used (bias-corrected-bootstrapped-test; 5000 replications, 95% Confidence Interval). Confidence Intervals with the lower and the upper bounds, both positive or both negative, were used to verify the significance of the effects [45].

Finally, to address *RQ4 (Moderating Effects*), Model 1 from Hayes-PROCESS-tool was used. The statistical significance of interaction effects was inspected (*p* < 0.05), the delta R-sq values (ΔR^2^) were reported to display that the inclusion of the interaction term resulted in a statistically significant increase in the variance explained in the outcome. Simple slopes were plotted to display moderating effects graphically. For diagnosing multicollinearity, the Variance Inflation Factor (VIF) and tolerance values were calculated and VIF < 5 and tolerance > 0.30 were used as cut-off points to identify multicollinearity issues [46,47,48]. All the statistical analyses were conducted by using SPSS, Version-21.

## 3. Results

### 3.1. Research Question One (RQ1)—Preliminary Differences in Study Variables

Responding to RQ1, data highlighted statistically significant differences between sampled nurses according to the working unit. Specifically, for background information, data revealed statistically significant differences in the sample composition, except for type of contract and performing night shifts. Considering sex, the majority of nurses working in ICU were men, while almost all of the nurses working in NICU were women (*p* < 0.001). Moreover, nurses working in ICU were significantly older (*p* < 0.001) and had higher working seniority (*p* < 0.05) than those working in NICU (Table 2).

Considering the study variables, firstly, Skewness (range −0.41 to +1.87) and Kurtosis values (range −0.77 to +4.70) indicated that data were approximately normally distributed. Therefore, comparing ICU and NICU nurses, data revealed they did not differ in terms of perceived levels of *Stressors in Nursing*, neither in terms of perceived stress related to *Death-and-Dying* nor in terms of the further stressors (*p* > 0.05 for all the comparisons).

Differently, nurses working in ICU perceived higher *Work-Resources* (with large effect sizes for both *Job-Control d* = 0.892, 95% CI [0.362 to 1.41] and *Rewards d* = 0.834, CI [0.308 to 1.35]) as well as lower *Psychological Disease* (with medium effect size *d* = 0.719, CI [−1.23 to −0.198]) than nurses working in NICU (Table 3).

Furthermore, considering clinically relevant levels of *Psychological Disease* identified by using the cut-off scores provided by the Italian validation study of the SCL-90-R [40], data showed that 14.3% of ICU nurses and 33.3% of NICU nurses reported clinically relevant psychological burden, yet no statistically significant differences between the working units was found (χ^2^ = 3.16; *p* = 0.07).

### 3.2. Research Question Two (RQ2)—Main Effects

Responding to RQ2, commonalities and specificities in the associations between perceived *Stressors in Nursing*, *Work-Resources*, and *Coping-Strategies*, respectively, with *Psychological Disease* according to the working unit were found (Table 4).

Specifically, perceived *Workload* and the recourse to *Self-Blame* and *Wishful Thinking* coping strategies (risk factors) and perceived *Rewards* (protective factors) emerged as common factors significantly associated with *Psychological Disease* across the working units (ICU and NICU).

Otherwise, perceived stress related to *Death-and-Dying* and *Conflicts-with-Supervisors*, and the recourse to *Seek-Advice* and *Escape/Avoidance* coping strategies emerged as specific risk factors significantly associated with *Psychological Disease* in nurses working in ICU.

Differently, perceived stress related to *Conflicts-with-Peers*, *Conflicts-with-Physicians*, and *Uncertainty-concerning-Treatment* (risk factors) and perceived levels of *Job-Control* and *Social-Support* (protective factors) emerged as specific factors significantly associated with *Psychological Disease* in nurses working in NICU.

### 3.3. Research Question Three (RQ3)—Mediating Effects

Preliminarily to testing mediating (*RQ3*) and moderating analyses (*RQ4*), Spearman’s rank correlation between *Background Information* and study variables were conducted according to the working unit to verify whether and which background factors should be included as control variables (Supplementary Table S1).

**Figure 2:**
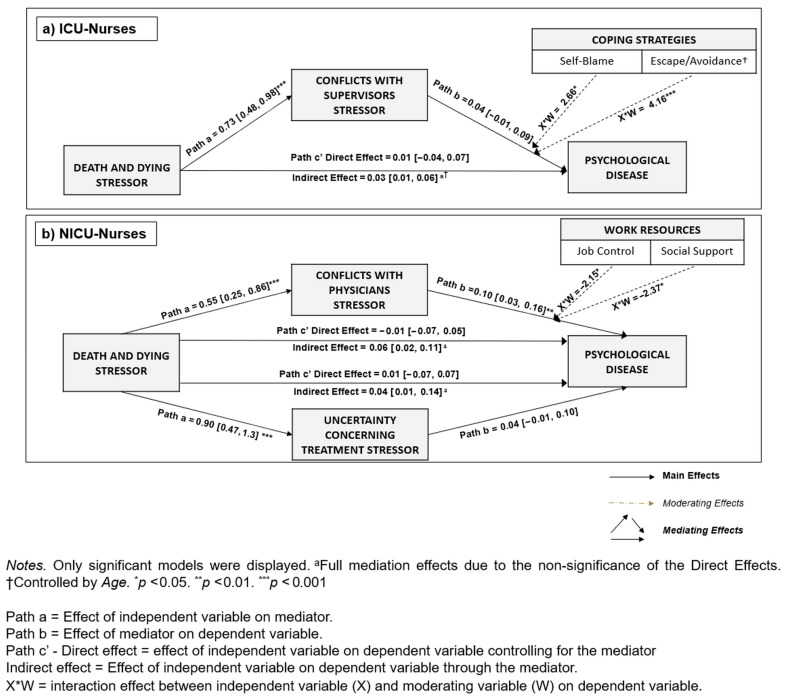
Summary: statistically significant mediating and moderating effects among (**a**) ICU nurses and (**b**) NICU nurses.

Therefore, responding to RQ3, data revealed that *Death-and-Dying*-*Stressor* was associated indirectly—through the association with specific *Stressors in Nursing*—with *Psychological Disease*. Also, these *Stressors in Nursing* varied according to the working units (Table 5; Figure 2). Specifically, for nurses working in ICU, after controlling for *Age* (due to the significant correlation between *Age* and *Death-and-Dying*-*Stressor r_s_* (33) = 0.43, *p <* 0.05; see Supplementary Table S1), data from mediation analyses revealed that *Death-and-Dying*-*Stressor* was associated indirectly with *Psychological Disease*, through the association with *Conflicts-with-Supervisors* (full mediation) (Table 5; Figure 2). Considering the diagnosis for multicollinearity, the Variance Inflation Factor (VIF) and tolerance values for the variables included in the mediation model (with *Psychological Disease* as outcome), namely *Age* (VIF = 1.25; Tolerance = 0.80), *Death-and-Dying*-*Stressor* (VIF = 2.52; Tolerance = 0.40), and *Conflicts-with-Supervisors* (VIF = 2.16; Tolerance = 0.46), indicated the lack of multicollinearity issues.

For nurses working in NICU, data from mediation analyses revealed that *Death-and-Dying-Stressor* was associated indirectly with *Psychological Disease*, through the association with both *Conflicts with Physicians* and *Uncertainty Concerning Treatment* (full mediations), respectively (Table 5; Figure 2). Also in such cases, the VIF and tolerance values for the variables included in the mediation models comprising *Conflicts-with-Physicians* (VIF = 1.56; Tolerance = 0.64) and *Uncertainty Concerning Treatment* (VIF = 1.73; Tolerance = 0.58) indicated the lack of multicollinearity issues. For the analyses carried out with the sampled nurses working in NICU, no background variables were included in the mediation models as control variables due to the lack of significant correlations with the relevant study variables underpinning the models (see Supplementary Table S1).

No other statistically significant mediating effects were found.

### 3.4. Research Question Four (RQ4)—Moderating Effects

**Figure 3:**
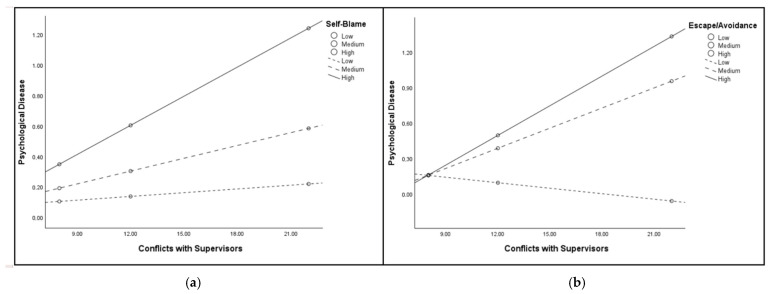
The moderating role of Self-Blame (**a**) and Escape/Avoidance (**b**) Coping Strategies in the associations between Conflicts with Supervisors and Psychological Disease among nurses working in ICU.

Responding to RQ4, data supported the moderating role of *Work-Resources* and *Coping-Strategies*, also highlighting specificities according to the working unit (Figure 2). Specifically, for nurses working in ICU, data revealed the statistically significant conditional effects of *Self-Blame* and *Escape/Avoidance* coping strategies (the latter model controlled by *Age* due to the significant correlation between *Age* and *Escape/Avoidance* coping strategies *r_s_*(33) = 0.40, *p* < 0.05; see Supplementary Table S1) in the associations between *Conflicts-with-Supervisors* and *Psychological Disease* (Table 6; Figure 3). Furthermore, the VIF and tolerance values for the variables included in the moderation models comprising *Self-Blame* (VIF = 1.04; Tolerance = 0.95) and *Escape/Avoidance* (*Age* VIF = 1.14; Tolerance = 0.87; *Conflicts-with-Supervisors* VIF = 1.08; Tolerance = 0.92; *Escape/Avoidance* VIF = 1.21; Tolerance = 0.83) indicated the lack of multicollinearity issues.

**Figure 4:**
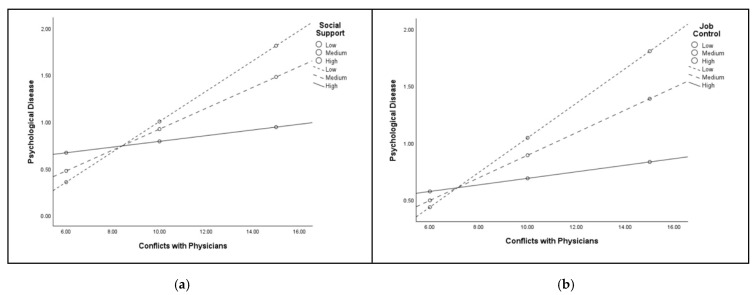
The moderating role of Social Support (**a**) and Job Control (**b**) in the associations between Conflicts with Physicians and Psychological Disease among nurses working in NICU.

For nurses working in NICU, data revealed the statistically significant conditional effects of *Job-Control* and *Social-Support* in the associations between *Conflicts-with-Physicians* and *Psychological Disease* (Table 6; Figure 4). Also in such cases, the VIF and tolerance values for the variables included in the moderation models comprising *Job-Control* (VIF = 1.16; Tolerance = 0.86) and *Social-Support* (VIF = 1.07; Tolerance = 0.93) indicated the lack of multicollinearity issues. As for mediation analyses, for the moderation analyses carried out with the sampled nurses working in NICU, no background variables were included as control variables due to the lack of significant correlations with the relevant study variables underpinning the models (see Supplementary Table S1).

No other statistically significant moderating effects were found.

## 4. Discussion

The study applied the *DRIVE-Nurses-Model* [28,29,30,31,32,33] to explore and compare the experience of nurses working in ICU and NICU, by mainly focusing on examining the mechanisms underlying the relationship between *Death-and-Dying*-*Stressor* and nurses’ psychological health. This is to identify tailored risk profiles and protective factors to be accounted for in defining interventions fostering wellbeing in such highly demanding work environments as Intensive Care Units.

Firstly, considering preliminary comparisons (RQ1), findings indicated that sampled nurses statistically differed in terms of sex distribution, i.e., the majority of nurses working in ICUs were men—somewhat reflecting the recent global efforts to increase the number of male nurses [49]—yet all but one of the nurses working in NICU were women. These data seem to add empirical evidence which endorse the presence of still-operating stereotyped views of gender-specific roles and specialties, attributing neonatal care to female nurses [50].

Additionally, still considering RQ1, data revealed that sampled nurses working in NICU were overall younger and less steady/experienced than those working in ICU. This can also be discussed by considering the data highlighting that sampled nurses working in NICU perceived lower work-resources and reported higher psychological disease than nurses working in ICU. Indeed, research has suggested that less experienced nurses working in Intensive Care Units reported higher levels of stress and psychological suffering than those with higher seniority, training, and experience [51,52,53].

However—somewhat in contrast with research suggesting higher burden and workload among nurses working in NICU if compared with other ICUs [4]—data revealed that sampled nurses did not differ in terms of perceived levels of *Stressors in Nursing,* not even for perceived stress related to *Death-and-Dying*. Nonetheless, findings on main/mediating/moderating effects (RQ2-RQ4) identified commonalities and specificities in risk and protective factors to be accounted for promoting adjustment processes and psychological wellbeing among nurses working in ICU and NICU.

Considering commonalities, data aligned with research warning about the high challenges and demands characterizing work life in ICUs [4,5,6,7,8,9,10,11], highlighting the detrimental role of perceived *Workload*, yet also revealing the protective role of perceived rewards/recognition within the work context [54,55,56]. Furthermore—interestingly and somewhat in contrast with the literature [25,26,27]—the potential burden of the relationship with patients and their families does not seem to play a role in influencing nurses’ psychological health in ICU or in NICU.

However, data also suggested the need to carefully consider the coping strategies adopted by nurses for dealing with such high levels of burden [28,29,30]. Indeed, considering the burden of working with critically ill, unstable, and precarious patients “on the edge between life and death”, the recourse by nurses to *Self-Blame* (i.e., dealing with stress by feeling disproportionate sense of responsibility and guilt) or, conversely, to *Wishful Thinking* strategies (i.e., relying on hope/detaching from reality) represented two opposite ways that could, however, equally impair nurses’ wellbeing and the quality of care provided in both ICU and NICU.

Otherwise, considering specificities by working units—for nurses working in ICU—data underlined the psychological costs of the detrimental interplay between perceived *Death-and-Dying-Stressor* and *Conflict with Supervisors*, so that perceived stress linked to dealing with end-of-life experiences may impair—and be exacerbated by—the perceived relationship with supervisors/managers (the latter experienced as a further source of stress rather than a source of support). This dynamic can also be reflected by discussing data showing that the recourse by ICU nurses to seeking-support coping strategies may be ineffective and even counterproductive. From this perspective, findings suggest carefully monitoring the coping profile of ICU nurses, since our data emphasized that nurses’ attempts to manage stress and conflicts by relying on *Self-blame* [31,57] or, on the opposite pole, on *Escape/Avoidance*—to detach from extremely burdening work-related experiences [23,58,59]—can only heighten their psychological suffering, potentially increasing the sense of isolation, frustration, and discomfort due to the perceived lack of support from supervisors.

Differently, considering nurses working in NICU, data underlined the psychological costs of perceived stress related to *Conflicts with Peers*, along with the detrimental interplay between *Death-and-Dying-Stressor* and perceived *Conflicts with Physicians* and *Uncertainty concerning Treatment*, respectively. However, whether data clearly indicated the need to foster communication and collaboration among NICU staff members, the negative role of perceived uncertainty could either reflect the higher perceived fragility of the “suspended” newborn (when compared to adult ill patients hanging between life and death). Yet this finding may also be due to our sample composition (i.e., comprising overall less experienced nurses than those working in ICU) [51,52,53].

Nonetheless, for NICU nurses, data also supported the protective role of work-resources [28,29,30,31,32,33,55], specifically highlighting that perceived *Job-Control* and *Social-Support* could overwhelm the detrimental effects of perceived interpersonal conflicts with physicians. In other words, findings suggested the possibility of fostering nurse–physician relationships, by enhancing nurses’ skill-discretion/autonomy, without neglecting the different—yet not foremost—role of physicians, and sustaining reciprocity, respect, and mutual support.

In summary, the application of the *DRIVE-Nurses-Model* to explore and compare the experience of nurses working in ICU and NICU provided preliminary evidence to advance the understanding of the mechanisms underlying the relationship between *Death-and-Dying*-Stressor—unavoidable source of stress repeatedly featuring nursing daily life—and nurses’ psychological health, suggesting tailored risk profiles and key protective factors to account for to provide nurses with the necessary resources for adjusting to their intrinsically challenging work-related duties and experiences.

### Limitations

Findings should be interpreted with caution due to some limitations. Firstly, the study was carried out with a cross-sectional design. Therefore, despite this design being considered efficient and appropriate for exploring our research questions [60], no inferences concerning the temporal associations between predictors and outcomes was made, and causality cannot be established. Therefore, future research could be designed with a longitudinal design to further explore the relationships featuring the complex dynamics that emerged (main/mediating/moderating effects). Secondly, since the use of self-report measures, the risk of social desirability bias could be high and common method variance cannot be ruled out. Nevertheless, although research has demonstrated that this limitation could not influence the validity of our findings [61], future research could also be designed to include a wider range of sources of data.

Moreover, although the population being studied is inherently small and narrow [62], since it is a defined and limited work group (nurses working in Intensive Care Units, rather than the nursing workforce), the sample consists of a convenience sample of nurses working exclusively in Intensive Care Units from five Hospitals of the Public Health Service in a restricted geographical area (Southern Italy)—and participating voluntarily—thus limiting the generalizability of research results. Further studies on larger/nationally representative samples—or comparative studies including ICUs from other countries—are needed to generalize these results. Nonetheless, the small sample size does not weaken the clear advantages of adopting a transactional multidimensional and statistically valid model, such as the *DRIVE-Nurses-Model*, to be used by researchers and healthcare managers to assess and monitor the psychological health conditions of nursing staff to define tailored evidence-based interventions accordingly. In this direction, due to the flexibility of the *DRIVE-Nurses-Model*-framework, future research could also include/test the role of further potential resources (e.g., personality characteristics; job satisfaction) able to counteract the psychological costs of *Death-and-Dying-Stressor* and foster nurses’ wellbeing. Furthermore, considering that this is a delicate situation “on the borderline between life and death”, our data did not show—from the nursing staff’s perspective alone—that ‘the management of patients and their families’ represents a source of stress impacting nurses’ psychological health. Further research could be extended to all the medical staff (including physicians) and family members, in order to identify potential similarities and differences in the management of death and the dying.

## 5. Conclusions

The application of the *DRIVE-Nurses-Model* [28,29,30,31,32,33] allowed for advancing knowledge on the experiences of nurses working in Intensive Care Units (ICU and NICU), who are routinely exposed to the burden of dealing with critically ill/unstable/dying patients. The study provides preliminary evidence on specific risks and resources to be accounted for when defining customized evidence-based training and programs to target and intervene on those aspects of nursing working life that are amenable to change/can be improved (rather than on unmodifiable aspects, i.e., dealing with death/dying patients), such as interpersonal relationships within the wards, technical preparedness, work-resources, along with providing coping strategies to deal with work-related stress.

Specifically, for nurses working in ICU, the findings highlighted the need to primarily consider and address the psychological risk linked to perceived lack of support from supervisors (i.e., perceived isolation), the recourse to excessive guilt/blaming and/or the defensive detachment from work-related experiences. Accordingly, interventions fostering adjustment processes and psychological health among nurses working in ICU should be implemented by mainly targeting the relationship with supervisors and fostering—at the same time—adaptive ways of managing conflicts within the wards and stress related to the burden of nursing work.

For nurses working in NICU, instead, findings highlighted the need to primarily consider the psychological risk linked to perceived lack of competence/training and the lack of perceived support from direct co-workers, thus providing specific training and opportunities for professional development, autonomy, and growth, yet also fostering a reciprocal/mutual supportive work environment that recognizes the voices of all the professionals in the management of critically ill newborns.

Overall, healthcare policymakers and hospital managers should consider these findings, thus openly recognizing the “invisible” burden of routinely working with death and the dying, which challenges nurses’ lives on a daily basis. This can be achieved by providing adequate evidence-based interventions that prevent the denial of such burden and the escalation of psychological suffering while fostering individual, relational, and organizational resources within the healthcare contexts. This can be facilitated through psycho-educational training (e.g., [63]), psychodynamic-oriented interventions (e.g., [64]), and integrated approaches, such as the “Psycho-Educational Defusing Intervention” (e.g., [65])—the latter of which has been proven to be effective for preventing trauma and sustaining recovery and wellbeing among healthcare workers belonging to Emergency and Critical Care Units— since they promote nurses’ awareness and knowledge along with their psychosocial competencies by building/strengthening relational resources, fostering peer-supporting (e.g., defusing), and boosting the recourse to adequate coping strategies for dealing with such highly emotionally demanding work environments as the healthcare context.

## Figures and Tables

**Table 1:** Description of dimensions, measures, and variables included in the present study.

DIMENSIONS	MEASURES	VARIABLES
**Background Information**	***Single item Questions*** (5 items)	*Sex* (Men = 0; Women = 1); *Age* (in years); *Working Seniority* (in years); *Type of Contract* (Fixed-term contract = 0; Permanent contract = 1); *Performing Night Shifts* (No = 0; Yes = 1)
**Stressors in Nursing**	***Expanded Nursing Stress Scale*** ENSS [34] 57 items on 4-point Likert scale (1 = “never stressful”, 2 = “occasionally stressful”, 3 = “frequently stressful”, 4 = “always stressful”; 0 = “does not apply” **^b^**) Nine subscales (*Death and Dying, Conflict with Physicians, Patients and their Families, Conflicts with Peers, Conflicts with Supervisors, Uncertainty Concerning Treatment, Inadequate Emotional Preparation,* *Discrimination, Workload*)	*Death and Dying* (7 items; α = 0.77) *Conflict with Physicians* (5 items, α = 0.74) *Patients and their Families* (8 items, α = 0.78) *Conflicts with Peers* (6 items, α = 0.87) *Conflicts with Supervisors* (7 items, α = 0.80) *Uncertainty Concerning Treatment* (9 items, α = 0.85) *Inadequate Emotional Preparation* (3 items, α = 0.68) *Discrimination* (3 items α = 0.87) *Workload* (9 items, α = 0.81).
**Work Resources** ^**a**^	***Job Content Questionnaire*** JCQ [35] 27 items on a 4-point Likert scale (from 0 = “Often” to 3 = “Never”) Three subscales (*Job Demands*, *Social Support*, and *Job Control*).	*Social Support* (4 items, α = 0.73) *Job Control* (14 items, α = 0.75)
	***Effort-Reward Imbalance Test*** ERI-Test [36,37] 17 items on a 5-point Likert scale (from 1 = “Disagree” to 5 = “Agree, and I am very distressed”) Two subscales (*Effort*, *Rewards*)	*Rewards* (11 items, α = 0.83)
**Coping Strategies**	***Ways of Coping Checklist-Revised*** WCCL-R [38] 42 items on a 4-point Likert scale (from 0 = “Never used” to 3 = “Always used”) Five subscales (*Problem-focused, Seek-Advice, Self-Blame, Wishful Thinking, Escape/Avoidance*)	*Problem-focused* (15 items, α = 0.80) *Seek-Advice* (6 items, α = 0.88) *Self-Blame* (3 items, α = 0.78) *Wishful Thinking* (8 items, α = 0.89) *Escape/Avoidance* (10 items, α = 0.72).
**Psychological Disease**	***Symptom Checklist 90-Revised*** SCL-90-R [39,40] 90 items on a 5-point Likert scale (from 0 = “Not at all” to 4 = “Extremely”) Nine subscales (*Depression, Anxiety, Phobic Anxiety, Obsessive Compulsive Symptoms, Somatization, Hostility, Interpersonal Sensitivity, Paranoid Ideation, Psychoticism*).	*Global Severity Index* ^c^ (Cronbach’s α = 0.98)

*Notes.* ^a^ In line with the *DRIVE-Nurses Model* (and also in order to avoid overlapping analyses and results for *Job Demands* and *Efforts*, respectively, with *Workload*—measured by the *ENSS*), “work-resources” were assessed by using the two subscales of *Social Support* and *Job Control* from the JCQ, and the *Rewards* subscale from the ERI-Test. ^b^ The option “0 = does not apply” was defined as a non-applicable value (calculation of the average value was performed excluding zero) to ensure that the data distinguished and reflected nurses’ lack of experience of that event rather than the lack of perceived stress linked to that experience. ^c^ Global Severity Index (GSI) is a Global Score given by the sum of all responses divided by 90 and it indicates both the number of symptoms and the intensity of the psychological disease. Clinical levels of psychological disease were calculated by using the cut-off scores for the GSI, that is, respectively, 0.97 for men and 1.24 for women. Cronbach’s α referred to the present study.

**Table 2:** Characteristics of study participants (*N* = 62) according to the working unit.

	Total Sample (*N* = 62)	ICU Nurses (*n* = 35)	NICU Nurses (*n* = 27)	Adjusted *p*-Values ^a^
**Sex**, *n* (%)				
Women	37 (59.7)	11 (31.4)	26 (96.3)	
Men	25 (40.3)	24 (68.6)	1 (3.7)	0.000 ***
**Age in years**, Mean ± *SD*	42.90 ± 9.9	47.71 ± 6.2	36.67 ± 10.4	0.000 ***
**Working Seniority in years**, Mean ± *SD*	16.97 ± 9.5	20.94 ± 5.8	11.96 ± 10.8	0.02 *
**Type of contract**, *n* (%)				
Fixed-term contract	4 (6.5)	0 (0.0)	4 (14.8)	
Permanent contract	58 (93.5)	35 (100.0)	23 (85.2)	0.32
**Night shifts**, *n* (%)				
No	3 (4.8)	1 (2.9)	2 (7.4)	
Yes	59 (95.2)	34 (97.1)	25 (92.6)	1.00

*Notes.* Differences are calculated by χ^2^-Analyses [*n* (%)] and Student’s t-test (Mean ± Standard deviations). ^a^ Adjusted *p*-values calculated via Holm–Bonferroni correction method. Statistically significant values (* *p* ≤ 0.05; *** *p* < 0.001) are highlighted in bold.

**Table 3:** Means and standard deviations of the study variables according to the working unit (*N* = 62).

	Total Sample (*N* = 62)	ICU Nurses (*n* = 35)	NICU Nurses (*n* = 27)	Adjusted *p*-Values ^a^	Cohen’s *d* ^b^	
	*M* ± *SD*	*M* ± *SD*	*M* ± *SD*		Effect Size	95% CI
**Stressors in Nursing**						
Death and Dying	14.66 ± 6.04	14.34 ± 6.96	15.07 ± 4.68	1.00	−0.120	[−0.622, 0.383]
Conflicts with Physicians	9.56 ± 4.29	9.08 ± 4.27	10.18 ± 4.31	1.00	−0.256	[−0.759, 0.249]
Patients and Families	15.79 ± 7.14	17.00 ± 7.51	14.22 ± 6.43	0.91	0.393	[−0.115, 0.898]
Conflicts with Peers	8.82 ± 5.34	8.03 ± 5.69	9.85 ± 4.75	1.00	−0.344	[−0.848, 0.164]
Conflicts with Supervisors	14.08 ± 6.18	13.20 ± 6.50	15.22 ± 5.66	1.00	−0.329	[−0.833, 0.178]
Uncertainty concerning treatment	18.40 ± 7.67	17.25 ± 8.40	19.89 ± 6.46	1.00	−0.345	[−0.849, 0.162]
Inadequate emotional preparation	5.48 ± 2.64	5.14 ± 3.07	5.93 ± 1.92	1.00	−0.297	[−0.801, 0.209]
Discrimination	3.79 ± 4.10	4.20 ± 4.21	3.25 ± 3.96	1.00	0.229	[−0.276, 0.732]
Workload	17.43 ± 7.16	17.00 ± 7.53	18.03 ± 6.77	1.00	−0.148	[−0.650, 0.356]
**Work Resources**						
Job Control	37.03 ± 6.47	39.34 ± 5.29	34.03 ± 6.72	0.02 *	0.892	[0.362, 1.41]
Social support	11.29 ± 2.95	11.37 ± 3.37	11.18 ± 2.35	1.00	0.073	[−0.435, 0.580]
Rewards	40.87 ± 8.84	43.85 ± 8.12	37.00 ± 8.33	0.02 *	0.834	[0.308, 1.35]
**Coping Strategies**						
Problem-focused	24.00 ± 6.55	25.34 ± 7.47	22.25 ± 4.69	0.52	0.480	[−0.031, 0.988]
Seek Advice	9.53 ± 4.15	8.85 ± 4.14	10.40 ± 4.09	0.96	−0.376	[−0.881, 0.132]
Self-blame	3.80 ± 2.07	3.65 ± 2.09	4.00 ± 2.06	1.00	−0.165	[−0.667, 0.339]
Wishful Thinking	9.41 ± 5.59	8.48 ± 5.47	10.63 ± 5.61	0.96	−0.387	[−0.893, 0.121]
Escape/Avoidance	9.53 ± 4.37	9.05 ± 4.30	10.14 ± 4.47	1.00	−0.249	[−0.752, 0.256]
**Psychological Disease**						
Global Severity Index	0.70 ± 0.73	0.48 ± 0.72	0.98 ± 0.66	0.05 *	−0.719	[−1.23, −0.198]

*Notes.* Differences are calculated by Student’s t-test (Mean ± Standard deviations). ^a^ Adjusted *p*-values are calculated via Holm–Bonferroni correction method. Statistically significant values (* *p* ≤ 0.05) are highlighted in bold. ^b^ Cohen’s *d* values and 95% Confidence Interval (CI).

**Table 4:** Inter-correlations between study variables according to the working unit (ICU and NICU).

	NICU	1	2	3	4	5	6	7	8	9	10	11	12	13	14	15	16	17	18
ICU																			
**Stressors in Nursing**																			
1. Death and Dying		1	0.60 **	0.59 **	0.34	0.48 *	0.65 **	0.66 **	0.42 *	0.15	−0.33	−0.32	−0.24	0.05	0.07	0.32	−0.02	−0.03	0.30
2. Conflicts with Physicians		0.86 **	1	0.66 **	0.76 **	0.65 **	0.81 **	0.56 **	0.42 *	0.60 **	−0.38 *	−0.26	−0.43 *	0.07	0.17	0.41 *	0.24	0.32	0.59 **
3. Patients and Families		0.80 **	0.81 **	1	0.60 **	0.64 **	0.70 **	0.24	0.55 **	0.61 **	−0.27	−0.06	−0.30	0.20	0.06	0.26	0.01	0.15	0.26
4. Conflicts with Peers		0.66 **	0.68 **	0.48 **	1	0.75 **	0.70 **	0.31	0.37	0.80 **	−0.46 *	−0.09	−0.48 *	0.34	0.33	0.42 *	0.37	0.35	0.60 **
5. Conflicts with Supervisors		0.72 **	0.56 **	0.67 **	0.40 *	1	0.79 **	0.26	0.42 *	0.83 **	−0.24	−0.09	−0.55 **	0.22	0.23	0.33	0.33	0.22	0.36
6. Uncertainty concerning treatment		0.90 **	0.89 **	0.79 **	0.68 **	0.53 **	1	0.52 **	0.44 *	0.66 **	−0.41 *	−0.13	−0.40 *	0.19	0.29	0.39 *	0.21	0.24	0.44 *
7. Inadequate emotional preparation		0.76 **	0.80 **	0.69 **	0.73 **	0.36 *	0.78 **	1	−0.05	0.10	−0.37	−0.40 *	−0.16	−0.01	0.07	0.14	0.09	0.07	0.37
8. Discrimination		0.44 **	0.45 **	0.27	0.36 *	0.38 *	0.39 *	0.25	1	0.26	0.09	0.28	−0.25	−0.34	0.46 *	0.40 *	−0.01	0.18	0.16
9. Workload		0.78 **	0.74 **	0.82 **	0.68 **	0.78 **	0.65 **	0.64 **	0.36 *	1	−0.28	−0.02	−0.56 **	0.26	0.16	0.31	0.41 *	0.37	0.48 *
**Work Resources**																			
10. Job Control		−0.15	−0.09	0.06	−0.31	−0.11	−0.06	−0.18	−0.27	−0.29	1	0.29	0.39 *	−0.08	−0.13	−0.43 *	−0.30	−0.22	−0.41 *
11. Social support		0.02	0.03	−0.06	−0.10	0.13	−0.10	−0.14	−0.12	0.06	0.29	1	0.24	0.43 *	0.04	−0.19	−0.30	0.10	−0.42 *
12. Rewards		−0.11	−0.10	−0.13	−0.12	−0.34 *	−0.08	−0.09	−0.34 *	−0.21	0.20	0.41 *	1	−0.14	−0.15	−0.54 **	−0.49 **	−0.54 **	−0.65 **
**Coping Strategies**																			
13. Problem-focused		−0.02	−0.17	−0.04	−0.01	0.15	−0.02	−0.13	−0.06	−0.07	0.15	−0.24	−0.41 *	1	0.51 **	0.31	0.07	0.04	0.11
14. Seek Advice		0.04	0.00	−0.00	0.15	0.01	0.05	0.08	−0.09	0.01	0.00	−0.11	−0.19	0.61 **	1	0.36	0.41 *	0.14	0.27
15. Self-Blame		0.22	0.23	0.19	0.16	0.21	0.30	0.17	0.11	0.18	−0.17	−0.18	−0.32	0.17	0.28	1	0.56 **	0.52 **	0.53 **
16. Wishful Thinking		0.38 *	0.37 *	0.26	0.35 *	0.34 *	0.31	0.32	0.24	0.43 *	−0.36 *	−0.03	−0.26	0.12	0.53 **	0.49 **	1	56 **	0.46 *
17. Escape/Avoidance		0.38 *	0.39 *	0.25	0.34 *	0.27	0.37 *	0.47 **	0.09	0.31	−0.17	0.03	−0.26	0.10	0.35 *	0.72 **	0.33	1	0.33
**Psychological Disease**																			
18. Global Severity Index		0.36 *	0.29	0.26	0.32	0.42 *	0.28	0.27	0.33	0.43 **	−0.32	−0.14	−0.34 *	0.19	0.40 *	0.56 **	0.55 **	0.62 **	1

*Note.* * *p* < 0.05; ** *p* < 0.01.

**Table 5:** Mediating analyses: path coefficients according to the working unit.

	Independent Variable (X)	Mediator (M)	Dependent Variable (Y)	Path a [95% C.I.]	Path b [95% C.I.]	Path c′ Direct Effect [95% C.I.]	Indirect Effect [95% C.I.]
**ICU** **Nurses †**	Death and Dying	Conflicts with Supervisors	Psychological Disease	0.73 [0.48, 0.98] ***	0.04 [−0.01, 0.09]	0.01 [−0.04, 0.07]	0.03 [0.01, 0.06] ^a^
**NICU** **Nurses**	Death and Dying	Conflicts with Physicians	Psychological Disease	0.55 [0.25, 0.86] ***	0.10 [0.03, 0.16] **	−0.01 [−0.07, 0.05]	0.06 [0.02, 0.11] ^a^
	Death and Dying	Uncertainty Concerning Treatment	Psychological Disease	0.90 [0.47, 1.3] ***	0.04 [−0.01, 0.10]	0.01 [−0.07, 0.07]	0.04 [0.01, 0.14] ^a^

*Notes.* Path a = Effect of independent variable on mediator; Path b = Effect of mediator on dependent variable; Path c′: Direct effect = effect of independent variable on dependent variable controlling for the mediator; Indirect effect = Effect of independent variable on dependent variable through the mediator. ^a^ Full mediation effects due to the non-significance of the path c′ (Direct Effects). † Model controlled by Age. Only significant mediation models are displayed. ** *p* < 0.01. *** *p* < 0.001.

**Table 6:** Moderating analyses: regression coefficients according to the working unit.

				Interaction Effects X*W							Model Summary		
	Independent Variable (X)	Moderator (W)	Dependent Variable (Y)	b	se	*t*	[95% C.I.]	*ΔR* ^2^	*F*	*p*	*R* ^2^	*F*	*p*
**ICU** **Nurses**	Conflicts with Supervisors	Self-Blame coping	Psychological Disease	0.02	0.01	2.66	[0.01, 0.03]	0.11	7.07	0.012 *	0.52	11.29	0.000 ***
	Conflicts with Supervisors	Escape/Avoidance coping †	Psychological Disease	0.01	0.00	4.16	[0.01, 0.02]	0.19	17.33	0.000 ***	0.67	15.17	0.000 ***
**NICU** **Nurses**	Conflicts with Physicians	Job Control	Psychological Disease	−0.01	0.00	−2.15	[−0.02, −0.01]	0.10	4.66	0.041 *	0.50	7.57	0.001 ***
	Conflicts with Physicians	Social Support	Psychological Disease	−0.02	0.01	−2.37	[−0.04, −0.01]	0.11	5.63	0.026 *	0.55	8.98	0.000 ***

*Notes.* X*W = interaction effect between independent variable (X) and moderating variable (W) on dependent variable (Y). † Model controlled by *Age*. Only significant models are displayed. * *p* < 0.05; *** *p* < 0.001.

## Data Availability

The data that support the results of this study are available from the corresponding author upon reasonable request.

## Supplementary Materials

The following supporting information can be downloaded at: https://www.mdpi.com/article/10.3390/healthcare13182265/s1, Supplementary Table S1. “Correlations between background information and study variables according to the working unit (ICU and NICU)”.

## Abbreviations

The following abbreviations are used in this manuscript:

DRIVE	Demands-Resources and Individual Effects Model
ICU	Intensive Care Unit
NICU	Neonatal Intensive Care Unit
