# Supplementary material for: The Psychological Impact of Dealing with Death and the Risk of Dying Among Nurses Working in ICU and NICU: Specificities in Mediating and Moderating Variables
Source: Healthcare (Basel). 2025 Sep 10;13(18):2265. doi: 10.3390/healthcare13182265 (PMC12469550; doi:10.3390/healthcare13182265)
Supplement: Supplementary file 1 [file healthcare-13-02265-s001.zip › healthcare-3760658-supplementary.pdf]

Supplementary Table S1. Correlations between background information and study variables according to the working unit (ICU and NICU)

Variables	ICU Nurses					NICU Nurses				
	Sex [a]	Age [b]	Working Seniority [c]	Type of Contract [d]†	Night shifts [e]	Sex [a]	Age [b]	Working Seniority [c]	Type of contract [d]	Night shifts [e]
**Stressors in Nursing**										
Death and Dying	−0.02	**0.43 ***	0.29	/	−0.02	0.33	−0.12	−0.16	0.16	0.10
Conflicts with Physicians	−0.05	**0.53 ****	**0.45****	/	−0.11	0.15	−0.08	−0.02	0.17	0.25
Patients and their Families	0.01	**0.43***	**0.37***	/	−0.18	0.20	−0.01	0.02	0.15	0.07
Conflicts with Peers	−0.07	0.31	0.28	/	0.12	0.11	−0.19	−0.07	0.07	0.10
Conflicts with Supervisors	−0.13	0.16	0.08	/	−0.13	0.00	−0.07	0.05	−0.01	−0.06
Uncertainty Concerning Treatment	0.10	**0.39***	0.19	/	−0.01	0.14	−0.25	−0.16	0.05	0.09
Inadequate Emotional Preparation	−0.05	**0.42***	0.**44****	/	−0.07	0.26	−0.07	−0.13	0.21	0.06
Discrimination	−0.07	0.01	−0.11	/	0.22	−0.01	−0.29	−0.23	0.17	0.13
Workload	−0.12	0.31	**0.35***	/	−0.16	−0.11	0.06	0.21	−0.16	−0.13
**Work Resources**										
Job Control	−0.00	0.19	0.07	/	−0.18	−0.25	0.01	0.08	−0.07	−0.04
Social Support	−0.31	0.11	0.09	/	−0.26	−0.19	−0.24	−0.26	0.06	0.07
Rewards	−0.13	0.10	−0.00	/	0.01	0.06	−0.03	−0.22	−0.05	−0.03
**Coping Strategies**										
Problem-Focused	0.18	−0.27	−0.25	/	−0.06	−0.25	−0.35	−0.31	0.24	−0.18
Seek Advice	**0.38***	−0.08	−0.05	/	−0.28	−0.08	**−0.62****	**−0.62****	0.18	0.06
Self-Blame	0.28	0.22	0.12	/	−0.09	−0.01	−0.15	−0.12	−0.24	0.07
Wishful Thinking	**0.40***	0.17	0.08	/	−0.28	0.19	−0.31	−0.23	−0.31	0.32
Escape/Avoidance	0.24	**0.40***	0.16	/	−0.27	0.05	−0.30	−0.15	−0.01	0.27
**Psychological Disease**										
Global Severity Index	0.28	0.05	0.11	/	−0.27	0.04	0.08	0.21	−0.03	0.24

Notes. [a] Sex (Men = 0; Women = 1); [b] Age (in years); [c] Working Seniority (in years); [d] Type of Contract (Fixed-term contact = 0; Permanent contact = 1); Performing [e] Night Shifts (No = 0; Yes = 1). **†** Values not calculated since the whole sample of nurses working in ICU had a Permanent contract**.** Statistically significant values (* $p \leqslant$ 05; ** p <.01) are highlighted in bold.
